# Interaction of zinc and IAA alleviate aluminum-induced damage on photosystems via promoting proton motive force and reducing proton gradient in alfalfa

**DOI:** 10.1186/s12870-020-02643-6

**Published:** 2020-09-18

**Authors:** Liantai Su, Jianping Xie, Wuwu Wen, Jiaojiao Li, Peng Zhou, Yuan An

**Affiliations:** 1 School of Agriculture and Biology, Shanghai Jiao Tong University, Shanghai, 200240 People’s Republic of China; 2 Key Laboratory of Urban Agriculture, Ministry of Agriculture, Shanghai, 201101 China

**Keywords:** Aluminum, Cyclic electron flow, IAA, Zinc, Proton motive force, Proton gradient

## Abstract

**Background:**

In acidic soils, aluminum (Al) competing with Zn results in Zn deficiency in plants. Zn is essential for auxin biosynthesis. Zn-mediated alleviation of Al toxicity has been rarely studied, the mechanism of Zn alleviation on Al-induced photoinhibition in photosystems remains unclear. The objective of this study was to investigate the effects of Zn and IAA on photosystems of Al-stressed alfalfa. Alfalfa seedlings with or without apical buds were exposed to 0 or100 μM AlCl_3_ combined with 0 or 50 μM ZnCl_2_, and then foliar spray with water or 6 mg L^− 1^ IAA.

**Results:**

Our results showed that Al stress significantly decreased plant growth rate, net photosynthetic rate (Pn), quantum yields and electron transfer rates of PSI and PSII. Exogenous application of Zn and IAA significantly alleviated the Al-induced negative effects on photosynthetic machinery, and an interaction of Zn and IAA played an important role in the alleviative effects. After removing apical buds of Al-stressed alfalfa seedlings, the values of *pmf*, g_H_^+^ and Y(II) under exogenous spraying IAA were significantly higher, and ΔpH_*pmf*_ was significantly lower in Zn addition than Al treatment alone, but the changes did not occur under none spraying IAA. The interaction of Zn and IAA directly increased Y(I), Y(II), ETRI and ETRII, and decreased O_2_^−^ content of Al-stressed seedlings. In addition, the transcriptome analysis showed that fourteen functionally noted genes classified into functional category of energy production and conversion were differentially expressed in leaves of alfalfa seedlings with and without apical buds.

**Conclusion:**

Our results suggest that the interaction of zinc and IAA alleviate aluminum-induced damage on photosystems via increasing *pmf* and decreasing ΔpH_*pmf*_ between lumen and stroma.

## Background

Aluminum toxicity is a major factor limiting crop production in acid soils [1]. The inhibition of photosynthetic apparatus, including light-harvesting photosystem II (PSII), photosystem I (PSI) and cytochrome b_6_f, is an important reason for the crop production limitation. PSII has three functional domains: antenna of chlorophyll (Chl), reaction center (RC) and oxygen evolving complex (OEC). The antenna absorbs and transfers photon energy to the reaction center where the excited state electrons from Chl a molecules (P680) and the OEC are transferred to a series of electron acceptors [2]. PSI catalyzes the light-driven electron transfer from plastocyanin to ferredoxin located in the stromal side [3]. The electron transport from PSII to PSI is tightly coupled with the generation of the thylakoid proton motive force (*pmf*), which consist of electric field (ΔΨ) and pH (ΔpH) gradients between lumen and stroma, and is the driving force for ATP synthesis in plants [4, 5]*.* It has been shown that Al stress could closure reaction centers (RCs) of PSII and PSI, impair light harvesting complex antennas of PSII (LHCII) and PSI (LHCI), reduce energy transfer from antennas to RCs, and inhibit electron transfer on the acceptor sides of PSI [4, 6]. These effects directly contribute to the reduction of net photosynthetic rate and plant growth under Al stress.

Zinc is an essential nutrient for plants, not only as a catalytic factor in enzymes, but also as a necessary structural component of proteins [7]. Zn normally presents in a high level in plants, and exerts as a cofactor of over 300 enzymes. A whole genome survey shows that 4–10% of all sequenced proteins from prokaryotes and eukaryotes contain Zn-binding domains [8]. Zn plays an important role in decreasing the harmful effects of abiotic stresses through scavenging ROS, retaining heavy metal in roots and keeping heavy metal concentration in mesophyll cells in non-toxic forms [9–13]. These damage symptoms initially appear in young leaves and meristems of plants due to the low mobility of Zn [14]. Zn deficiency has been one of the most widespread micronutrient constraints in plants.

Auxin is one major hormone involved in plant adaptation to abiotic stresses. Singh and Prasad [15] reported that IAA improved photosynthetic efficiency of Cd-treated seedlings by restoring functional and structural attributes of photochemistry system. Al stress inhibits the IAA synthesis in alfalfa (*Medicago sativa* L), but this inhibition can be alleviated by Zn addition [16] due to zinc participating the IAA synthesis [17]. Thus, the poor photosynthetic performance due to Zn deficiency under Al stress may relate to the decrease of IAA synthesis. However, how auxin and Zn affecting Al-induced inhibition of photosynthetic efficiency is barely known.

Zn availability in acidic soils is generally high, thus, it easily leaches from acidic soil, and its absorption intensively antagonizes by other cations such as Al, ammonium and potassium. In acidic soils, Al competes with Zn to bind in its binding sites on plasma membrane of roots, consequently interferes with Zn uptake and causes Zn deficiency in plants [18]. Thus, plants often suffer from Zn deficiency in acidic soils. Lin et al. [19] reported that the presence of Zn at physiological concentrations could protect the cells by preventing the Al-induced superoxide generation in rice and tobacco. To our knowledge, Zn-mediated alleviation of Al toxicity is rarely studied, and the mechanism of Zn alleviating Al-induced photoinhibition in photosystems remains unclear. Because Zn participates in IAA synthesis, and both of Zn and IAA have the function of alleviating plant Al toxicity in acidic soils, we speculated that an interaction of Zn and IAA might occur in plants to affect photosynthetic apparatus under Al stress. Thus, we focused on studying the effects of Zn, IAA and their interaction on photosystems of plants under Al stress using alfalfa (*Medicago sativa* L*.*) as plant materials.

## Results

### IAA contents in shoots

**Fig. 1:**
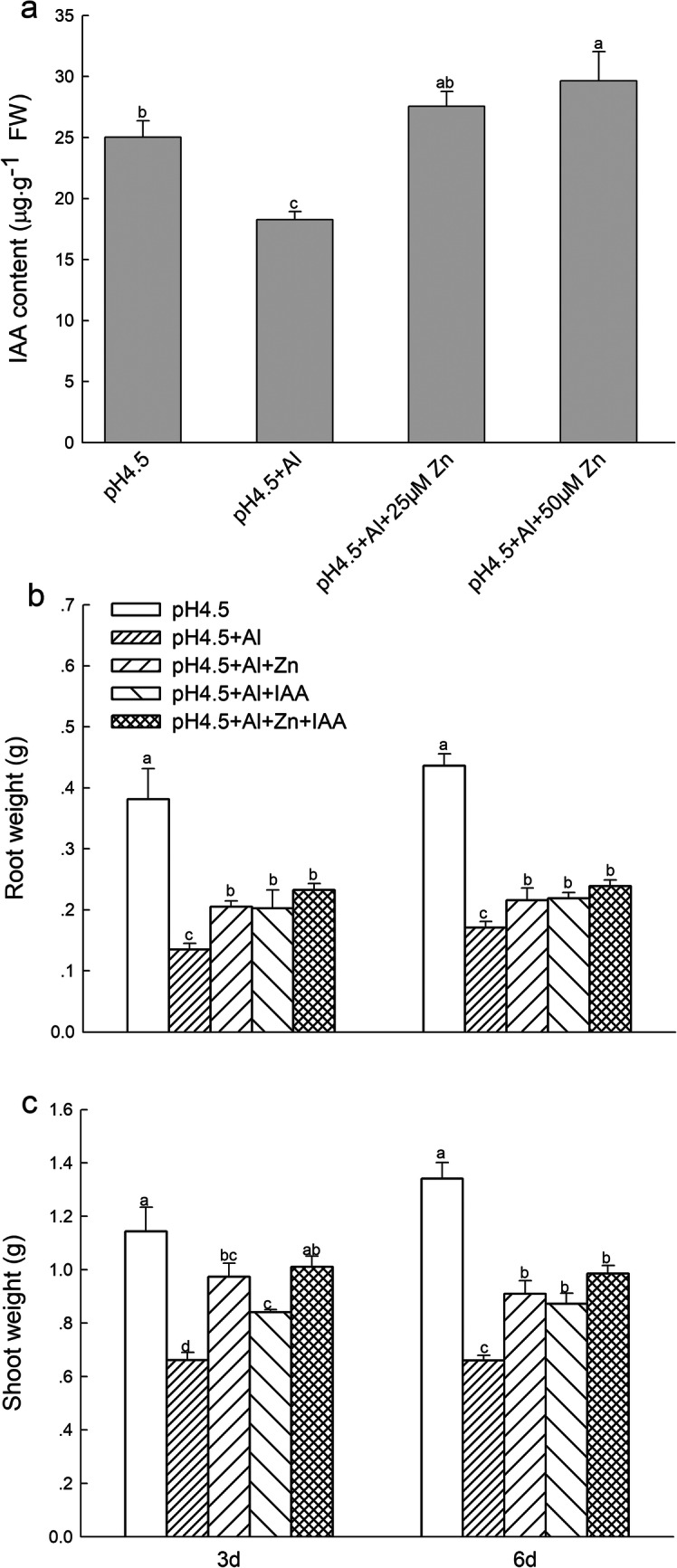
IAA contents in shoots (**a**) of alfalfa seedlings with apical buds grown in 1.5 mM Ca(NO_3_)_2_ medium (pH 4.5) containing 0 μM AlCl_3_ (pH 4.5), 100 μM AlCl_3_ (pH 4.5 + Al), 100 μM AlCl_3_ and 25 μM ZnCl_2_ (pH 4.5 + Al + 25 μM Zn), or 100 μM AlCl_3_ and 50 μM ZnCl_2_ (pH 4.5 + Al + 50 μM Zn) on 3 d. Fresh weights of root (**b**) and shoot (**c**) of alfalfa seedlings grown in 1.5 mM Ca(NO_3_)_2_ mediums (pH 4.5) containing 0 μM AlCl_3_ (pH 4.5), 100 μM AlCl_3_ (pH 4.5 + Al), 100 μM AlCl_3_ and 50 μM ZnCl_2_ (pH 4.5 + Al + Zn), 100 μM AlCl_3_ and 6 mg L^− 1^ IAA (foliar spray) (pH 4.5 + Al + IAA) or 100 μM AlCl_3_ and 50 μM ZnCl_2_ and 6 mg L^− 1^ IAA (foliar spray) (pH 4.5 + Al + Zn + IAA). Data are means ± SE of three replicates from three independent experiments. Bars with different letters indicate significant difference at *P* < 0.05 (Leas significant difference test)

IAA content in shoots was 37.0% lower in Al treatment than in control treatment, but addition of 25 μM or 50 μM Zn significantly increased IAA contents by 50.7 and 62.2%, respectively, in comparison with excess Al treatment (Fig.1a).

### Plant growth

Al stress significantly decreased fresh weights of roots (Fig.1b) and shoots (Fig. 1c), as well as root length (Additional file 1: Figure S1) compared with control treatment. The Al-induced growth inhibition was significantly alleviated after Zn and IAA applications either alone or combination, and the weights were significantly higher in the combined application of Zn and IAA than Zn or IAA application alone. There were no significant difference of root weights and shoot weights, separately, at 3rd and 6rd days after application of Zn and IAA under Al stress. Thus, the seedlings at 3rd day were mainly selected for the following study.

### Contents of Al and Zn in roots and shoots

Zn addition decreased Al contents in roots (Additional file 2: Figure S2a) and shoots (Additional file 2: Figure S2b) compared with Al treatment alone. Combined application of Zn and IAA further decreased Al contents in roots compared with Zn addition alone, but there was no significant difference in shoots between Zn application with or without IAA. Application of IAA alone increased Al contents in roots compared with Al treatment alone, but there was no significant difference in shoots.

Zn contents in roots (Additional file 2: Figure S2c) and shoots (Additional file 2: Figure S2d) were significantly higher in combined application of Zn and IAA than Zn addition under Al stress. The ratio of Al/Zn in roots and shoots was 7.88 and 0.63 in Zn addition, and 5.34 and 0.42 in combined application of Zn and IAA under Al stress, respectively (Additional file 2: Figure S2e, f). A lower Al/Zn ratio in combined application of Zn and IAA indicates that the interaction of Zn and IAA maintains Al/Zn homeostasis under Al stress condition.

### Photosynthetic rates, RuBisCO activities and carbonic anhydrase activities

**Fig. 2:**
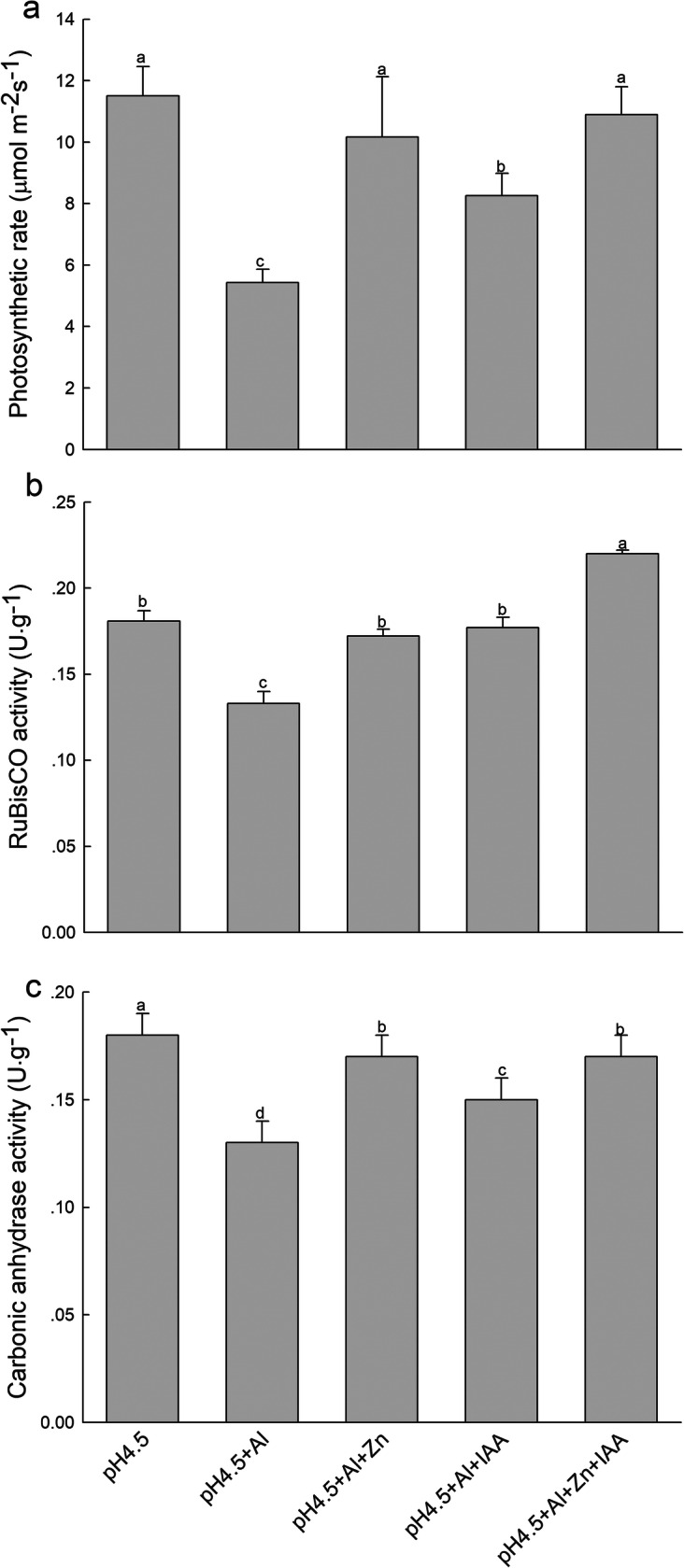
Net photosynthetic rate (**a**), RuBisCO activities (**b**) and carbonic anhydrase activities (**c**) in leaves of alfalfa seedlings with apical buds grown in 1.5 mM Ca(NO_3_)_2_ medium (pH 4.5) containing 0 μM AlCl_3_ (pH 4.5), 100 μM AlCl_3_ (pH 4.5 + Al), 100 μM AlCl_3_ and 50 μM ZnCl_2_ (pH 4.5 + Al + Zn), 100 μM AlCl_3_ and 6 mg L^− 1^ IAA (foliar spray) (pH 4.5 + Al + IAA) or 100 μM AlCl_3_ and 50 μM ZnCl_2_ and 6 mg L^− 1^ IAA (foliar spray) (pH 4.5 + Al + Zn + IAA) on 3 days. Data are means ± SE of three replicates from three independent experiments. Bars with different letters indicate significant difference at *P* < 0.05 (Leas significant difference test)

Net photosynthetic rates (Pn) of alfalfa seedlings significantly decreased under Al stress compared with control treatment, but application of Zn and IAA either alone or combination significantly increased the Pn compared with Al treatment alone. The Pn was lower in IAA application alone than Zn application with or without IAA (Fig.2a). Al stress significantly decreased foliar RuBisCO activity (Fig.2b) and foliar carbonic anhydrase activity (Fig.2c) in comparison with control treatment, while application of Zn and IAA either alone or combination significantly increased the two enzyme activities compared with Al treatment alone.

### Chlorophyll fluorescence parameters of PSI and PSII

**Fig. 3:**
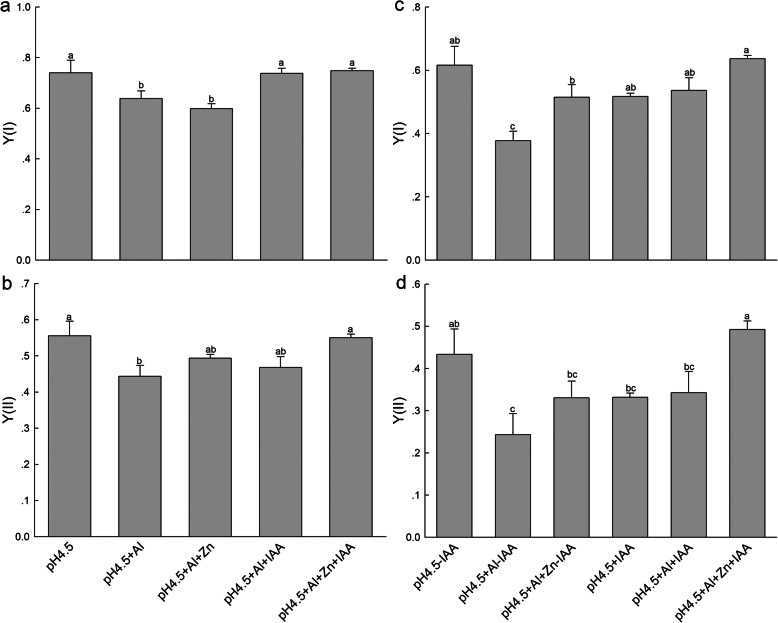
Light intensity dependence of photosynthetic quantum yields of PSI [Y(I)] and PSII [(Y(II)] in leaves of alfalfa seedlings with or without apical buds. Five treatments in the seedlings with apical buds are as Fig.2, and seedlings without apical buds are grown in 1.5 mM Ca(NO_3_)_2_ medium (pH 4.5) and treated with or without spraying IAA (pH 4.5-IAA, pH 4.5 + IAA), 100 μM AlCl_3_ with or without spraying IAA (pH 4.5 + Al-IAA, pH 4.5 + Al + IAA) and 100 μM AlCl_3_ and 50 μM ZnCl_2_ with or without spraying IAA (pH 4.5 + Al + Zn-IAA, pH 4.5 + Al + Zn + IAA). The Y(I) (**a**) and Y(II) (**b**) were estimated from seedlings with apical buds, and Y(I) (**c**) and Y(II) (**d**) were estimated from seedlings without apical buds on day 3. At least 6 different leaves from different seedlings were used for each treatment and data are means ± SE of three replicates. Bars with different letters indicate significant difference at *P* < 0.05 (Leas significant difference test)

Al stress significantly decreased effective quantum yields of PSI [Y(I)] and PSII [Y(II)] compared with control treatment, while application of IAA and Zn in combination significantly increased the Y(I) (Fig.3a) and Y(II) (Fig.3b) compared with Al treatment alone. The Y(ND), Y(NA), Y(NPQ) and Y(NO) were shown in Additional file 3: Figure S3a-d.

After removing apical buds of alfalfa seedlings, Al stress significantly decreased the Y(I) (Fig.3c) and Y(II) (Fig.3d) under none spraying IAA compared with control treatment. Zn addition significantly increased Y(I) under none spraying IAA and Y(II) under spraying IAA compared with Al treatment. Both Y(I) and Y(II) were higher under spraying IAA than none spraying IAA under Al stress. The Y(ND), Y(NA), Y(NPQ) and Y(NO) were shown in Additional file 3: Figure S3e-h.

The number and area of yellow-green spot in leaves of seedlings with apical buds were larger in Al treatment alone than Zn and IAA addition either alone or combination, indicating that Al stress decreased maximum quantum yield of primary photochemistry (Fv/Fm). Application of Zn and IAA greatly alleviated Al induced damage on primary photochemistry (Additional file 4: Figure S4).

### Light intensity dependence of photosynthetic electron transport rates in PSI and PSII

**Fig. 4:**
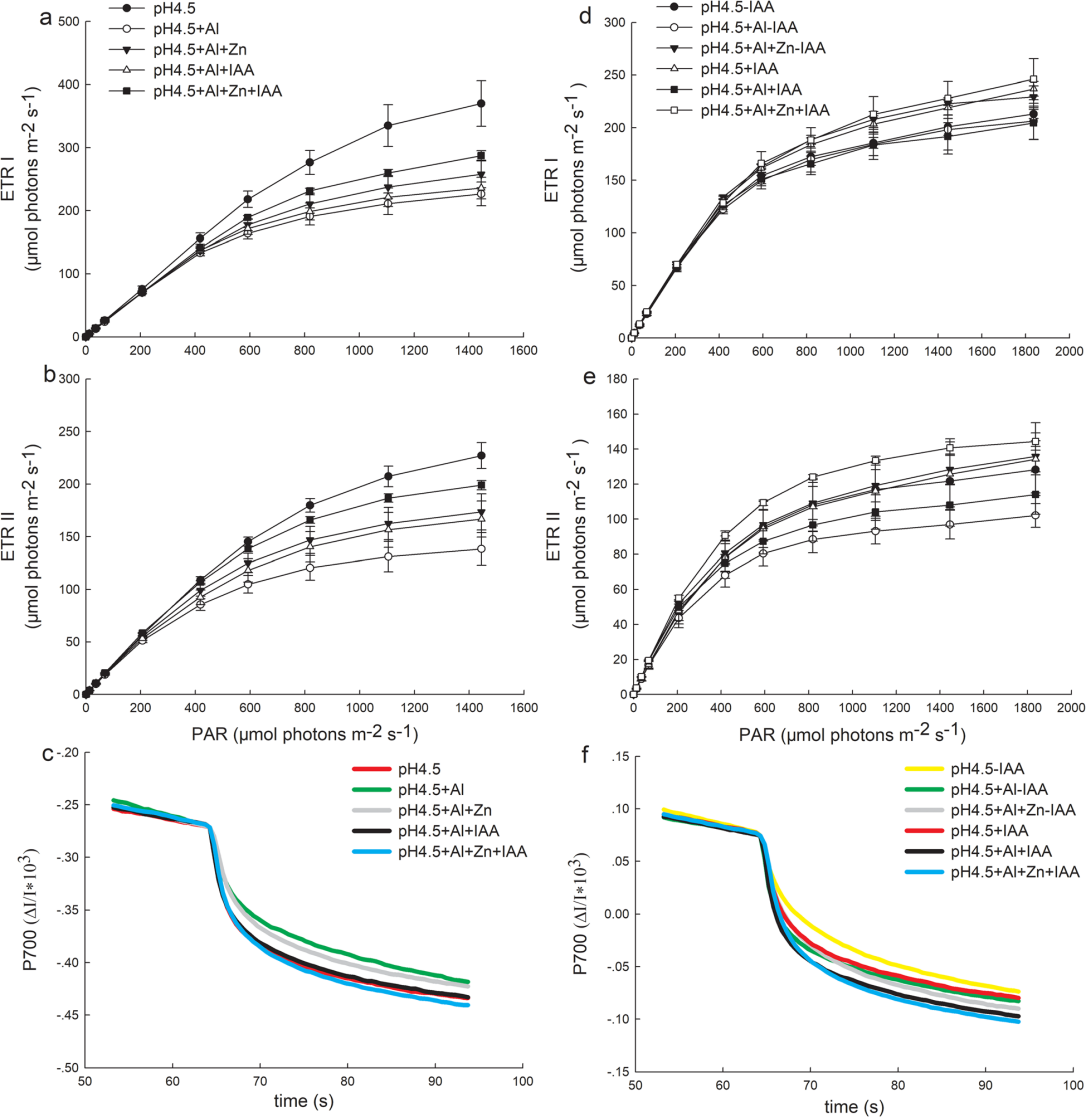
Light intensity dependence of the photosynthetic electron flow through PSI (ETRI) and PSII (ETRII), and P700^+^ reduction curve in leaves of alfalfa seedlings with or without apical buds. Five treatments in the seedlings with apical buds are as Fig.2, and six treatments in the seedlings without apical buds are as Fig.3. The ETRI (**a**), ETRII (**b**) and P700^+^ reduction curve (**c**) were estimated from seedlings with apical buds, and ETRI (**d**), ETRII (**e**) and P700^+^ reduction curve (**f**) were estimated from seedlings without apical buds on day 3. At least 6 different leaves from different seedlings were used for each treatment and data are means ± SE of three replicates

Al stress strongly inhibited ETRI and ETRII, and their initial slopes of curves were lowest among all treatments (Fig.4). Applications of Zn and IAA either alone or combination alleviated the Al-induced decreases of ETRI (Fig.4a) and ETRII (Fig.4b), and the ETRI and ETRII were higher in combined application of Zn and IAA than Zn or IAA application alone.

Application of Zn and IAA either alone or combination greatly increased the initial slopes of P700^+^ reduction curves compared with Al treatment alone, which means that the releasing rate of electrons from oxidized P700 was increased by Zn and IAA addition, and the releasing rate was higher in the combined application of Zn and IAA than Zn or IAA application alone (Fig.4c).

After removing apical buds of alfalfa seedlings, Al stress greatly inhibited ETRI (Fig.4d) and ETRII (Fig.4e) under both spray and none spray of IAA. Zn addition with or without spray IAA greatly increased the ETRI and ETRII of Al-stressed seedlings, with higher increases of ETRI and ETRII under spraying IAA. The initial slopes of the P700^+^ reduction curves were higher under spraying IAA than none spraying IAA, and the highest initial slope occurred in Zn treatment with spraying IAA among the six treatments (Fig.4f).

The minimum saturating irradiances (I_*k*_) of PSI and PSII were significantly decreased by Al stress, and Zn and IAA addition either alone or combination significantly increased I_*k*_ of PSI (Additional file 5: Figure S5a) and PSII (Additional file 5: Figure S5b) compared with Al stress.

### Cyclic electron flow

**Fig. 5:**
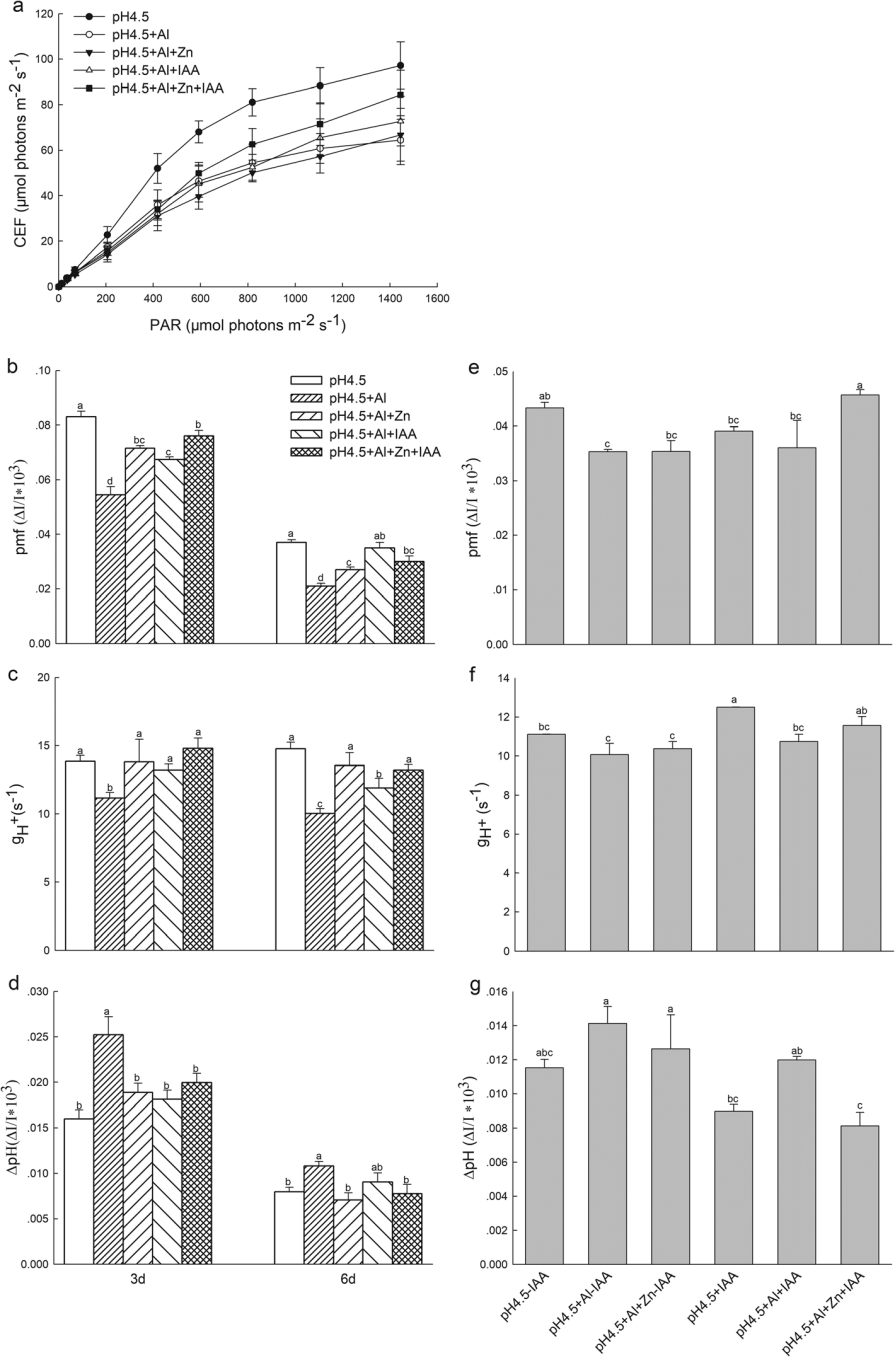
Cyclic electron flow around PSI (CEF) and parameters derived from the dark-interval relaxation kinetic of ECS (DIRK_ECS_) in the seedlings with or without apical buds. Five treatments in the seedlings with apical buds are as Fig.2, and six treatments in the seedlings without apical buds are as Fig.3. CEF (**a**), total proton motive force (*pmf*) (**b**), the proton gradient (ΔpH) (**c**) and the thylakoid proton conductivity (g_H_^+^) (**d**) were estimated from seedlings with apical buds, and *pmf* (**e**), ΔpH (**f**) and g_H_^+^ (**g**) were estimated from seedlings without apical buds. At least 6 different leaves from different seedlings were used for each treatment and data are means of three replicates. Bars with different letters indicate significant difference at *P* < 0.05 (Leas significant difference test)

The light response change of cyclic electron flow (CEF) around PSI was decreased after exposure to excess Al in comparison with control, but application of Zn and IAA either alone or combination greatly increased CEF under excess Al, and the highest increase was under the combined treatment of Zn and IAA (Fig.5a).

### Proton motive force and thylakoid proton conductivity

At light intensities of 582 μmol photons m^− 2^ s^− 1^, the total proton motive force (*pmf*) (Fig.5b) and proton conductivity (g_H_^+^) (Fig.5c) in thylakoid membrane significantly decreased under Al stress compared with control treatments. Application of Zn and IAA either alone or combination significantly alleviated the negative effects, and increased their *pmf* and g_H_^+^ under Al stress. Both of *pmf* and g_H_^+^ were higher in the combined application of Zn and IAA than Zn or IAA application alone. Excess Al significantly increased ΔpH_*pmf*_ compared with control treatment, but application of Zn and IAA either alone or combination reduced the ΔpH_*pmf*_ compared with Al treatment alone (Fig.5d).

After removing apical buds of alfalfa seedlings, Al stress significantly decreased *pmf* (Fig.5e), but did not affect g_H_^+^ (Fig.5f) and ΔpH_*pmf*_ (Fig.5g) compared with control treatment under none spraying IAA. Meanwhile, Zn addition did not affect the *pmf*, g_H_^+^ and ΔpH_*pmf*_ compared with Al treatment. Under condition of spraying IAA, Al stress significantly decreased g_H_^+^ compared with control treatment, but Zn addition increased *pmf* and decreased ΔpH_*pmf*_ compared with Al treatment. The values of *pmf* and g_H_^+^ under Al stress either alone or combination with Zn were lower under none spraying IAA than spraying IAA, while ΔpH_*pmf*_ was higher under none spraying IAA than spraying IAA.

### Activation state of ATP synthase and H^+^-ATPase activity

**Fig. 6:**
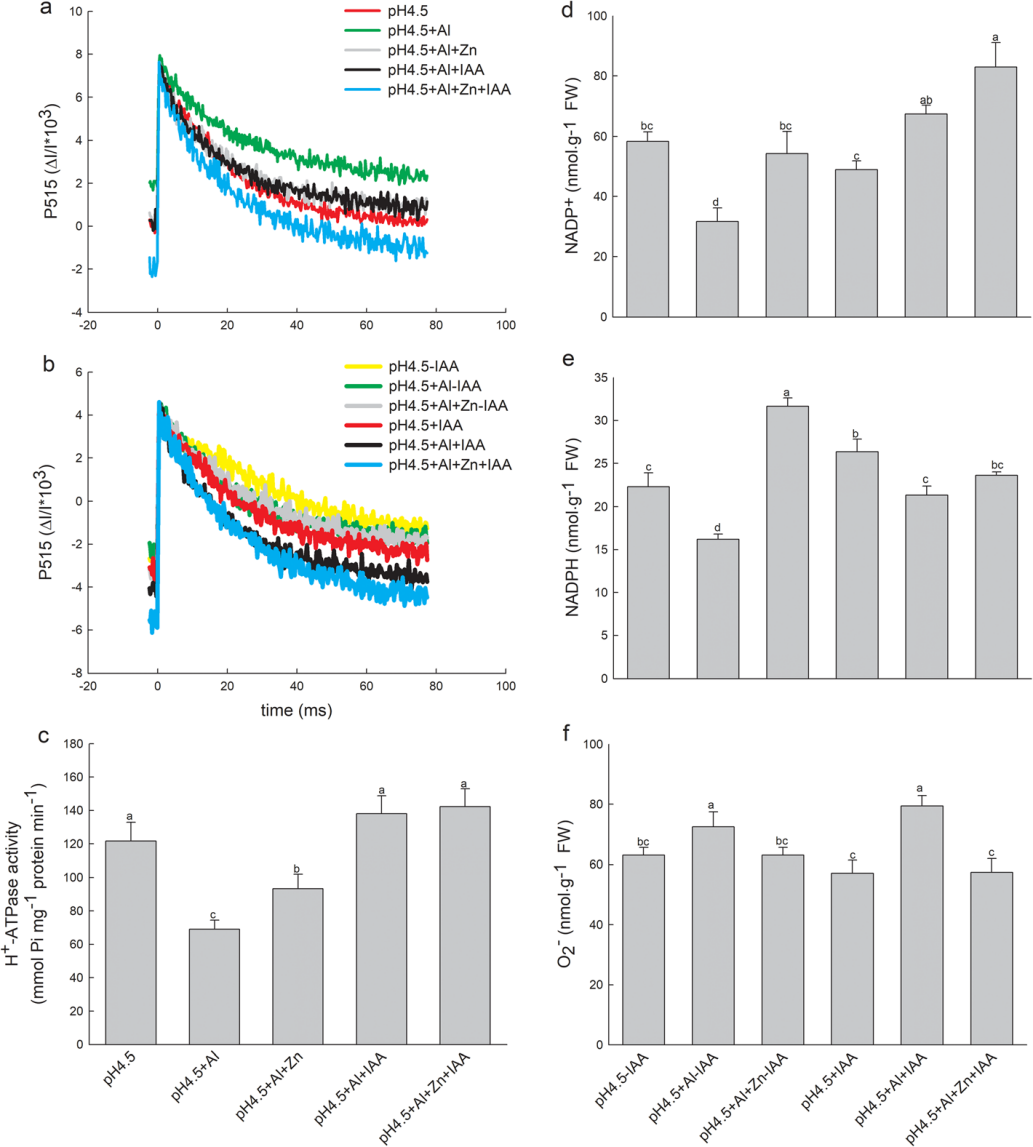
Changes of P515 signal in leaves of alfalfa seedlings with apical buds (**a**) and without apical buds (**b**), and H^+^-ATPase activity in shoots (**c**) of alfalfa seedlings with apical buds. Contents of NADP^+^ (**d**), NADPH (**e**) and O_2_^−^ (**f**) were measured in leaves of alfalfa seedlings without apical buds. Five treatments in the seedlings with apical buds are as Fig.2. Six treatments in the seedlings without apical buds are as Fig.3. All the data were measured on day 3. Data are means ± SE of three independent replicates. Bars with different letters indicate significant difference at *P* < 0.05 (Leas significant difference test)

Changes in P515 signals reflect the activation state of ATP synthase located in thylakoid membrane [20]. The initial slop of P515 curve is positive to the activation state of the ATP synthase. The ATP synthase activities were lowest in excess Al treatment alone, and highest in the combined treatment of Zn and IAA (Fig.6a). After removing apical buds of alfalfa seedlings, the ATP synthase activities were higher under spraying IAA than none spraying IAA (Fig.6b). The combined application of Zn and IAA had highest ATP synthase activities among all treatments.

Excess of Al significantly decreased foliar H^+^-ATPase activity in comparison with control treatment, while application of Zn and IAA either alone or combination significantly increased H^+^-ATPase activities in Al-stressed alfalfa seedlings, and IAA application with or without Zn addition had higher H^+^-ATPase activities than Zn treatment alone (Fig.6c).

### Contents of NADP^+^, NADPH and O_2_^−^

After removing apical buds, NADP^+^ contents significantly decreased under none spraying IAA, but increased under spraying IAA compared with control treatments in the presence of Al. Zn addition increased NADP^+^ contents of Al-stressed seedlings either with or without introduction of IAA (Fig.6d). NADPH contents significantly decreased under both with or without introduction of IAA in the presence of Al, while Zn addition significantly increased NADPH content of Al-stressed seedlings under none spraying IAA (Fig.6e). Al stress increased O_2_^−^ contents compared with control treatments, but significantly decreased by Zn addition comparted with Al treatments under both with or without introduction of IAA (Fig.6f).

### Transcriptome of alfalfa leaves after removed apical buds

The effect of removing apical buds on transcriptome of alfalfa leaves was investigated using RNA-seq.

**Fig. 7:**
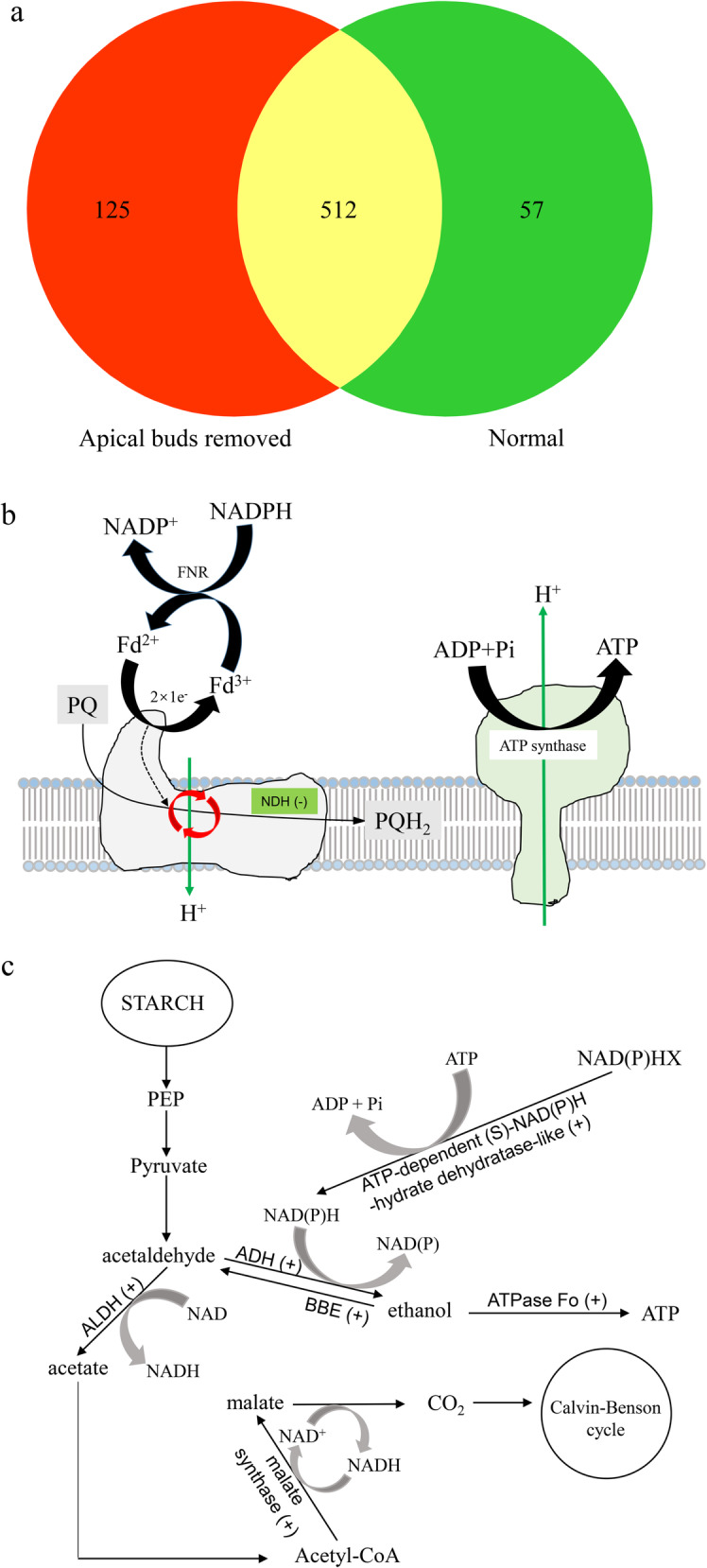
The genes related to energy production and conversion in RNA-seq assays from leaves of alfalfa seedlings with or without apical buds. **a** the number of genes involved in energy production and conversion was 694 in total, 57 and 125 only expressed in normal and apical buds removed seedlings, respectively, and 512 expressed in both normal and apical buds removed seedlings. The possible pathways in which DEGs involved were shown in (**b**) & (**c**), − represented downregulated genes, + represented upregulated genes. The gene_ids were listed in Additional file 7: Table S2

We focused on the genes classified into functional category of energy production and conversion. The genes in this category were most related to electron transport and energy pathways, and the number of genes involved in energy production and conversion was 694 in total (11 of them were upregulated and 3 of them were downregulated compared with normal seedlings, |log2(FC)| > =1 & *p*-value < 0.05). The complete list of differentially expressed genes (DEGs), their annotation, *p* values, and fold changes, was provided as supplementary material (Additional file 6: Table S1). Among the 694 genes, 57 and 125 only expressed in normal and apical buds removed seedlings, respectively, and 512 expressed in both normal and apical buds removed seedlings (Fig.7a). Fourteen DEGs (eleven were upregulated and three were downregulated) related to energy production and conversion were functionally annotated with eggNOG (Additional file 7: Table S2) and their possible pathways were shown in Fig.7b, c.

## Discussion

As a catalytic or structural cofactor of a large number of enzymes, Zn plays an important role in metabolic progresses related to photosynthesis and photochemistry. Carbonic anhydrase (CA) is a Zn-containing enzyme that catalyzes the reversible conversion of CO_2_ to HCO_3_ [21]. Application of Zn enhances the CA activity, which facilitates the supply of CO_2_ from the stomatal cavity to the site of CO_2_ fixation, and leads to Pn increase in pistachio seedlings under salt stress [9]. In the present study, application of Zn significantly increased the CA activity, as well as RuBisCO activity, leading to an increase of Pn under Al stress condition.

There are two electron transfer pathways related to proton motive force (*pmf*) formation: linear electron flow and cyclic electron flow (CEF). The CEF can reduce electron accumulation in the acceptor side of PSI by oxidizing the acceptor-side components of PSI, and then regulate *pmf* and proton gradient (ΔpH) of thylakoid. In the process, electrons recycle from PSI to plastoquinone (PQ) pool and Cytochrome *b*_*6*_*f* rather than to O_2_ to form ROS [22, 23]*.* In the present study, Zn addition increased CEF values of Al-stressed alfalfa, which increased *pmf* and proton transfer from lumen to stroma (g_H_^+^), as well as decreased ROS generation (Fig.6f). The generation of *pmf* links to ATP synthesis and balances ATP/NADPH ratio [22, 24]. The increased *pmf* and g_H_^+^ in Al-stressed seedlings activated chloroplastic ATP synthase located in thylakoid membrane (Fig.6a), consequently increased ATP synthesis accompanying with H^+^ transfer from lumen to stroma, which leads to a reduction of ΔpH_*pmf*_ between lumen and stroma.

The ΔpH_*pmf*_ formation controls modulation of PSII antenna light harvesting “switch”, and negatively regulates the electron transfer from PSII to PSI [25]. Thus, the down-regulated CEF-dependent generation of ΔpH_*pmf*_ by Zn addition prevented the Al-induced reduction of oxidized state of quinone QA and reaction centers of photosystems, and increased abilities of PSI and PSII to endure high light intensity (I_*k*_) (Additional file 5: Figre S5a, b). Meanwhile, the down-regulated ΔpH_*pmf*_ by Zn addition increased the amount of active population of P700 (Fig.4c, f), which led to a high capacity of electron carriers in PSI and a high electron transfer from oxidized P700 to alternative electron acceptors of PSI, consequently, enhanced ETRII and ETRI.

In the photosynthetic process, H^+^-ATPase extrudes proton to create an electrochemical gradient (proton gradient, ΔpH) between the lumen and stroma by consuming ATP, which regulates electron transfer in photosystems and finally affects CO_2_ assimilation [16, 26, 27]. Auxin functions as a systemic signaling compound affecting H^+^ transfer in cells and H^+^ secretion from roots by activating plasma membrane (PM) H^+^-ATPase [28, 29]. Thus, IAA and H^+^-ATPase play an important role in regulating membrane potential and proton gradient (ΔpH)*.* Zn is essential for auxin biosynthesis [30, 31]. Our previous study showed that excess Al significantly decreased the IAA concentrations in leaves and root tips of alfalfa. However, this negative effect was alleviated by Zn addition [16]. In addition, exogenous application of IAA significantly increased PM H^+^-ATPase activity and H^+^ secretion from root tips of alfalfa under Al stress [32]. These results indicate that Zn alleviates the Al-induced damage on photosynthetic machinery may be related to the increase of IAA synthesis and its effect on photosystems. An interaction between Zn and IAA might participate in alleviating Al-induced inhibition on photosynthetic machinery. Thus, we further studied the interactive effect of Zn and IAA on regulating photosystems of Al-stressed alfalfa seedlings with or without apical buds by exogenous application of IAA.

Exogenous application of IAA, in combine with Zn, increased *pmf* compared with Al stress alone, as well as compared with Zn or IAA addition alone under Al stress. The increased *pmf*, combined with the highest H^+^-ATPase activity, promoted ATP synthase activity on thylakoid membrane (Fig.6a) and increased ATP synthesis and H^+^ transfer from lumen to stroma [32]. These would ameliorate H^+^ environment in cells of Al-stressed seedlings, and led to highest quantum yields of Y(II) and Y(I) among the five treatments, accounted for 74.8 and 55.5% of the total excitation energy in PSI and PSII, respectively. The increased quantum yields effectively decreased the amount of non-photochemical energy dissipation and provided enough excited state energy to activate reaction centers and energy transfer to PSII and PSI, rather than energy transfer from chlorophyll to oxygen, resulting in less ROS production (Fig.6f). In addition, the highest values of active population of P700 (Fig.4c) and CEF (Fig.5a) were observed in Zn + IAA treatment under Al stress, indicating a low level of electron accumulation in PSI, which would protect P700 and increase ETRII and ETRI. These results implies that the interaction of Zn and IAA greatly alleviated the Al toxicity on photosynthetic machinery.

Our previous study showed that most of IAA was synthesized in apical buds of alfalfa. Removing apical buds would cause IAA concentration decrease to a very low level within 24 h [16]. The obvious phenomenon of IAA natural decrease after removing apical buds was used in present study to further test the interactive effect of Zn and IAA on photosystems of Al-stressed alfalfa seedlings. In the study, the apical buds were removed and the seedlings were sprayed with water or IAA. Under none spraying IAA, the values of Y(II), *pmf*, ΔpH_*pmf*_ and g_H_^+^ were not significantly different after Zn addition compared with Al treatment alone, however, Zn addition significantly increased *pmf*, and decreased ΔpH_*pmf*_ after spraying IAA. Meanwhile, Y(I), Y(II), ETRI and ETRII also significantly increased under the treatment, and they were highest among all six treatments. The removing apical bud study clearly demonstrates that the interaction of Zn and IAA directly affects *pmf* and ΔpH_*pmf*_ formation under Al stress, thus, affects proton and electron transfer in cells.

Zn can interfere with ROS generation produced by the membrane-bound NADPH oxidase [33], which depends on *pmf* and H^+^-ATPase. In the removing apical bud study, the content of O_2_^−^ significantly decreased after Zn addition compared with Al treatments, and the lowest content of O_2_^−^ occurred in Zn addition combined with spraying IAA (Fig.6f), indicating that the increased *pmf* regulates more electron transfer to generate NADPH rather than to generate ROS under Al stress. Concomitantly, this interaction induced increases of RuBisCO activity (Fig. 2b) and CO_2_ assimilation (Pn) would consume much of linear electron flow products (ATP and NADPH), which led to a reduction of NADPH accumulation and an increase of NADP^+^ accumulation. These would further promote electron transfer to NADPH generation rather than to ROS generation in chloroplast under Al stress. Thus, the interaction of Zn and IAA alleviated Al-induced photoinhibition in photosystems may greatly attribute to decreasing ROS generation.

Cyclic electron flow (CEF) around PSI produces only ATP, with no accumulation of NAD(P)H. In the process, two partially redundant pathways are involved. The major pathway is mediated by a complex containing PROTON GRADIENT REGULA-TION 5 (PGR5) and PGR5-Like1 (PGRL1). The minor pathway depends on the activity of the chloroplast NADH dehydrogenase-like (NDH) complex, a homolog to respiratory Complex I. This pathway is thought to prevent over-reduction of the chloroplast stroma in C_3_ plants, especially under abiotic stress conditions [34], where the NDH pumps approximately two protons from the chloroplast stroma to lumen per electron transferred from ferredoxin to plastoquinone, which effectively increases the efficiency of ATP production via CEF [35]. The coupling of proton and electron transfer reactions within NDH allows electron transfer from plastoquinol to NADPH to be driven by the thylakoid *pmf* [36]. In the present study, transcriptome analysis demonstrated that the *NADH dehydrogenase* gene was down-regulated in the leaves of removed apical bud seedlings, which might reduce electron transfer from ferredoxin to plastoquinone, and decrease ATP production. The decreased *NADH* expression was consistent with the decrease of P515 signal in leaves of removed apical bud seedlings. In plants, Zeaxanthin enhances NPQ induction in bright light. By contrast, a low-light environment causes a decrease in the proton gradient that activates the enzyme zeaxanthin epoxidase (ZEP), this converts zeaxanthin back into violaxanthin and causes a decrease in NPQ induction. The down-regulated *ZEP* expression in removed apical bud seedlings would keep a higher level of zeaxanthin and enhance NPQ induction, this would contribute to redundant energy dissipation in PSII and decrease electron transfer from PSII to PSI for photoprotection. These conclusions support the results of decreased ETR II and ETR I observed in removed apical bud seedlings. The above results of transcriptome analysis fully support our conclusion that IAA enhances Al tolerance of alfalfa via increasing *pmf* and decreasing ΔpH_*pmf*_ between lumen and stroma.

The effect of IAA on carbon fixation and energy production can also be clearly reflected from molecular regulation pathways of energy production and conversion. In plants, anaerobic activation of fermentative pathways constitutes an efficient solution to restore the pool of oxidized NAD^+^, while at the same time, avoiding pyruvate and succinate accumulation. Two fermentative pathways are expected to contribute mainly to glycolysis maintenance in plants: lactate and ethanol fermentation [37]. In the pathways, pyruvate is catalyzed by pyruvate decarboxylase (PDC) and a zinc-binding alcohol dehydrogenase (ADH), using NAD^+^ or NADP^+^ as a co-factor, and generates acetaldehyde and ethanol [38]. The ethanol either in allylic or benzylic form also can be oxidized to the corresponding aldehydes by berberine bridge enzyme (BBE) [39]. Aldehydes are highly reactive molecules, and are toxic at high concentrations. Aldehyde dehydrogenases (ALDHs), mitochondrial or cytosolic homotetrameric enzymes, oxidize acetaldehyde into acetate for biosynthesis of acetyl-CoA and malate, using NAD^+^ or NADP^+^ as a co-factor. As such ALDHs play an important role in detoxifying acetaldehyde in plants [38]. In the present study, *PEPC*, *ADH*, *malate synthase* (*MS*), two *ALDH* genes (*ALDH2-B7, C5*) and three *BBE* genes (*BBE8, 17 and 18*) were up-regulated in the leaves of removed apical bud seedlings, these might increase productions of ethanol, acetate and malate, while at the same time, the up-regulated *BBEs* genes would avoid more ethanol accumulation to damage cells. Ethanol synthesis, accompanied with an up-regulated *ATP synthase F0* gene in the leaves of removed apical bud seedlings, allowed glycolysis to continue producing a small quantity of ATP [40]. Furthermore, up-regulated *ATP-dependent (S)-NAD(P)H-hydrate dehydratase-like* gene, a metabolite repair or metabolite-proofreading enzyme, in the leaves of removed apical bud seedlings might convert abnormal metabolite NAD(P)H hydrate (NAD(P)HX) to NAD(P)H to restore the pool of NADH [41]. Thus, as a feedback response of IAA shortage in the seedlings of removed apical buds, ethanol synthesis and oxidation, as well as up-regulation of *ATP-dependent (S)-NAD(P)H-hydrate dehydratase* gene helped to remediate mismatches of ATP and NAD(P)H in the photosynthetic budget under down-regulated *NADH dehydrogenase* in the leaves of removed apical bud seedlings.

## Conclusion

Al stress significantly damaged photosynthetic apparatus of alfalfa, however, application of Zn and IAA significantly alleviated the Al-induced negative effects on photosystems. The interaction of Zn and IAA significantly increased quantum yields and electron transfer rates of PSI and PSII under Al stress. These positive effects were greatly attributed to the increases of *pmf*, H^+^-ATPase activity and ATP synthase activity, and the decrease of ΔpH_*pmf*_ between lumen and stroma. As a result, more electrons transferred to NADPH generation rather than to ROS generation, which greatly protected photosystems against excess Al damage.

## Methods

### Plant materials and growth conditions

Alfalfa seeds (WL-525HQ), which obtained from the Chinese National Seed Group Corporation, Ltd., germinated on a filter paper moistened with ½-strength Hoagland’s nutrient solution at 25 °C. The uniform seedlings were transplanted to a foam board (12 holes/board; 6 seedlings/hole) floating on aerated ½-strength Hoagland’s nutrient solution (pH 5.8) in plastic containers for 4 d. The seedlings were placed in greenhouse, and conditions set to 25/20 °C (day/night), 14-h photoperiod and a photon flux density of 400 μmol m^− 2^ s^− 1^. Solutions were replaced every 2 d.

### Treatments and experimental design

Our previous study showed that Al stress reduced the contents of Zn, Ca, Mg, Mn and K in roots of alfalfa seedlings grown in ½-strength Hoagland’s nutrient solution [42], but increased Fe content. The Al-induced imbalance of cation levels in plants will make us difficulty to distinguish the effect of Zn on alleviation of Al-induced photosystem damage. To avoid the imbalance of cation levels to disturb our identification on Zn’s effect on photosystem of alfalfa seedlings in Hoagland’s nutrient solution, a simple solution (1.5 mM Ca(NO_3_)_2_) was used in this study according to Sun et al. [43].

Experimental one: The interaction of Zn and IAA on photosystems of alfalfa seedlings with apical buds under Al stress. The dosage of 50 μM Zn was used in the experiments according to our preliminary experiments (Additional file 1: Figure S1). The alfalfa seedlings were placed in 1.5 mM Ca(NO_3_)_2_ solution (pH 4.5) supplemented with 0 μM AlCl_3_ (pH 4.5), or 100 μM AlCl_3_ with or without spraying 6 mg L^− 1^ IAA (pH 4.5 + Al, pH 4.5 + Al + IAA), or 100 μM AlCl_3_ and 50 μM ZnCl_2_ with or without spraying 6 mg L^− 1^ IAA (pH 4.5 + Al + Zn, pH 4.5 + Al + Zn + IAA).

In addition, the seedlings were treated with 1.5 mM Ca(NO_3_)_2_ solution (pH 4.5) supplemented with 100 μM AlCl_3_ (Al) either alone or in combined with 25 μM or 50 μM ZnCl_2_ to observe the variation of IAA contents in seedlings. The 1.5 mM Ca(NO_3_)_2_ solution with pH 4.5 was used as control treatment.

Experimental two: The interaction of Zn and IAA on photosystems of alfalfa seedlings without apical buds under Al stress. Most of IAA were synthetized in apical bud of alfalfa, and removing apical buds greatly decreased the IAA content in alfalfa [16]. To further explore the interaction of Zn and IAA on protecting photosystems under Al stress, a removing apical bud experiment was conducted. After 3 d of apical buds removed, the seedlings were placed in 1.5 mM Ca(NO_3_)_2_ solution (pH 4.5) supplemented with 0 μM AlCl_3_ with or without spraying IAA (pH 4.5-IAA, pH 4.5 + IAA), or 100 μM AlCl_3_ with or without spraying IAA (pH 4.5 + Al-IAA, pH 4.5 + Al + IAA) or 100 μM AlCl_3_ and 50 μM ZnCl_2_ with or without spraying IAA (pH 4.5 + Al + Zn-IAA, pH 4.5 + Al + Zn + IAA).

Each treatment in the above experiments was replicated three times and all measurements or plant sample collection were conducted at the 3rd day.

### Measurement of gas exchange, chlorophyll fluorescence and P700 parameters

Five leaves per treatment were used for measurements of net photosynthetic rate (P_n_) with GFS-3000 (Walz, Germany) under the light intensity at 800 μmol m^− 2^ s^− 1^ and air flow rate at 750 μmol s^− 1^. The measurements were conducted three times independently.

Chlorophyll fluorescence and P700 parameters were measured simultaneously by Dual-PAM-100 system (Walz, Germany). Prior to measurements, all plants were in the dark for more than 2 h, and fluorescence induced curve (Slow Kinetics) was determined in ‘Fluo+P700’ mode. Then, the kinetics of chlorophyll fluorescence induction and P700 oxidation were recorded simultaneously from the instrument. The light-adapted photosynthetic parameters were recorded after exposure to different light intensity (1445, 1105, 819, 592, 418, 206, 69, 36, 13 μmol photons m^− 2^ s^− 1^) for 240 s. The chlorophyll fluorescence parameters *Fv/Fm, Y*(*II*), *Y*(*NPQ*) and *Y*(*NO*) were calculated as described in Huang et al. [44]. P700 parameters were measured and calculated as described in Yuan et al. [45] and Huang et al. [46].

Photosynthetic electron flow through PSI or PSII were calculated as: ETRII=Y(II) × PPFD× 0.84 × 0.5, ETRI=Y(I) × PPFD× 0.84 × 0.5 [47]. The value of cyclic electron flow (CEF) was estimated as ETRI-ETRII [44]. Chlorophyll fluorescence images of treated leaves were measured at room temperature with an imaging PAM (Imaging WinGigE, Walz, Germany) after dark-adapting plants for 1 h, according to procedures described in Xia et al. [48].

I_*k*_ is minimum saturating irradiance and reflects tolerance ability of photosystems to high light intensity. The value of I_*k*_ was estimated using the empirical equation of rapid light curve (RLC) proposed by Eilers and Peeters [49].

Total proton motive force (*pmf*), g_H_^+^ and proton gradient (ΔpH) across the thylakoid membranes were estimated from the total amplitude of the rapid decay of the ECS signal as described in Li et al. [50] and Huang et al. [44]. Alfalfa seedlings were first dark-adapted for 1 h before ECS signal was detected. The P515/P535 was measured according to Zhang et al. [51] and Li et al. [50], and the ATPase activity was evaluated from P515 changes induced by saturating single turnover flashes.

With regard to the above-mentioned experiments, at least 6 different leaves from different seedlings were used for each treatment.

### Assay of RuBisCO activity, carbonic anhydrase activity, H^+^-ATPase activity, and contents of IAA, NADP^+^, NADPH, superoxide anion (O_2_^−^), Al and Zn

The activities of RuBisCO, carbonic anhydrase and plasma membrane H^+^-ATPase were measured using commercial enzyme-linked immunoassay (ELISA) kits according to the manufacturer’s instruction (JL22727 & JL22872 & JL49554, Jianglai biotech, China). Briefly, about 1 g fresh shoots from different treatments were homogenized in 9 mL cold phosphate buffer (PBS, 0.01 M, pH 7.40) on ice. The homogenates were then centrifuged at 5000×g for 10 min at 4 °C. The extract (supernatants) from plants captures the antibody and encapsulates the antibody onto the micropore plate to make the solid phase antibody. Then, the samples (RuBisCO, carbonic anhydrase or H^+^-ATPase) were added to the encapsulated micropore and combined with the labeled antibody to form the antibody antigen-enzyme-labeled antibody complex. After a thorough washing, the substrate TMB was added and colored. The color is positively correlated with the activities of RuBisCO, carbonic anhydrase or H^+^-ATPase. Finally, the absorbance was determined immediately at 450 nm, and the activities of these enzymes were calculated with a standard curve.

The contents of NADP^+^ and NADPH were determined according to the manufacturer’s instruction (Cominbio, Suzhou, China). Briefly, about 0.1 g fresh shoots from different treatments was ground into homogenate with 1 mL acidic extracting solution (for NADP^+^) or 1 mL alkaline extracting solution (for NADPH) in a mortar on ice. The homogenate was then transferred to a 1.5 mL Eppendorf tube and immersed in a water-bath at 95 °C for 5 min, quickly cooled in ice bath and centrifuged at 10000×g for 10 min at 4 °C. The 500 μL supernatant was collected in a new tube and another 500 μL alkaline extracting solution (for NADP^+^) or 500 μL acidic extracting solution (for NADPH) were added to neutralize, then mixed and centrifuged at 10000×g for 10 min at 4 °C. The supernatants were collected for analysis.

Al and Zn contents were determined according to Wang et al. [32] with minor modification. Briefly, fresh samples from treated plants were oven-dried for 72 h at 80 °C, and then grounded to fine powder. A 0.5 g powder was digested in a 1:1 (v/v) nitric acid/hydrogen peroxide solution (HNO_3_/H_2_O_2_). Al and Zn contents were then determined using an inductively coupled plasma emission spectrometer (ICP-AES; Iris Advantage 1000, Jarrell Ash Corp. Franklin, Massachusetts, USA).

IAA content was determined following Wang et al. [16], and O_2_^−^ content was measured according to the manufacturer’s instruction (Cominbio, Suzhou, China), fresh samples were used and the extraction of O_2_^−^ were conducted at low temperature (4 °C or in an ice bath). Three biological repeats were performed for each of above experiment.

### RNA-seq analysis

After part of alfalfa seedlings (12 d from germination) were removed apical buds for 48 h, the total RNA samples were isolated from the alfalfa seedlings with or without apical buds by using *TransZol Up Plus RNA Kit* (TransGen Biotech. China) following the manufacturer’s instruction. The quality control was performed using an Agilent 4200 Bioanalyzer (Agilent Technologies Inc.). To analyze the transcriptome, RNASeq libraries were prepared from cDNA by Instrumental Analysis Center (Shanghai Jiao Tong University, Shanghai, China) and sequenced on an Illumina NovaSeq 6000 (Illumina Inc., San Diego, CA, USA). Raw sequencing reads were screened with the FASTP software (Version 0.20.0) to cut out low quality or default reads. All gained clean reads were assembled to Transcripts or Unigenes using TRINITY software (Version 2.8.5). Gene expression (FPKM) and differential expression levels were analyzed using RSEM and edgeR software. We gained gene function annotation from the assembled Unigenes using BLAST and diamond software (Version 0.8.22) with the NCBI-NR (nonredundant) database, Swiss-Prot, eggNOG, GO and KEGG.

### Statistical analysis

All of the treatments were repeated three times, and the data were assessed from the results of three independent experiments. Data was analyzed by variance (ANOVA) using SAS 9.0 (SAS Institute Inc., Cary, NC, USA) and the means were compared by least significant difference (LSD) at *P* = 0.05 level. Different letters on the histograms indicate statistical differences at the level of *P* < 0.05.

## Supplementary information

**Additional file 1 Figure S1.** Root length of alfalfa seedlings grown in 1.5 mM Ca(NO_3_)_2_ medium (pH 4.5) containing 0 μM AlCl_3_ (pH 4.5), 100 μM AlCl_3_ (pH 4.5 + Al), 100 μM AlCl_3_ and 25 μM ZnCl_2_ (pH 4.5 + Al + 25 μM Zn), or 100 μM AlCl_3_ and 50 μM ZnCl_2_ (pH 4.5 + Al + 50 μM Zn) on days 1, 3 and 6. Data are means ± SE of three replicates from three independent experiments. Bars with different letters in the same day indicate significant difference at *P* < 0.05 (Leas significant difference test).

**Additional file 2 Figure S2.** Al contents in roots (a) and shoots (b), Zn contents in roots (c) and shoots (d) and Al/Zn ratio in roots (e) and shoots (f) of alfalfa seedlings with apical buds grown in 1.5 mM Ca(NO_3_)_2_ medium (pH 4.5) containing 0 μM AlCl_3_ (pH 4.5), 100 μM AlCl_3_ (pH 4.5 + Al), 100 μM AlCl_3_ and 50 μM ZnCl_2_ (pH 4.5 + Al + Zn), 100 μM AlCl_3_ and 6 mg L^− 1^ IAA (foliar spray) (pH 4.5 + Al + IAA) or 100 μM AlCl_3_ and 50 μM ZnCl_2_ and 6 mg L^− 1^ IAA (foliar spray) (pH 4.5 + Al + Zn + IAA) on 3 days. Data are means ± SE of three replicates from three independent experiments. Bars with different letters indicate significant difference at *P* < 0.05 (Leas significant difference test).

**Additional file 3 Figure S3.** Light intensity dependence of photosynthetic quantum yields of Y(ND) and Y(NA) in PSI and Y(NPQ) and Y(NO) in PSII in leaves of alfalfa seedlings with or without apical buds. Five treatments in the seedlings with apical buds are as Fig.3, and seedlings without apical buds are grown in 1.5 mM Ca(NO_3_)_2_ medium (pH 4.5) and treated with or without spraying IAA (pH 4.5-IAA, pH 4.5 + IAA), 100 μM AlCl_3_ with or without spraying IAA (pH 4.5 + Al-IAA, pH 4.5 + Al + IAA) and 100 μM AlCl_3_ and 50 μM ZnCl_2_ with or without spraying IAA (pH 4.5 + Al + Zn-IAA, pH 4.5 + Al + Zn + IAA). The (a) Y(ND), (b) Y(NA), (c) Y(NPQ) and (d) Y(NO) were estimated from seedlings with apical buds, and (e) Y(ND), (f) Y(NA), (g) Y(NPQ) and (h) Y(NO) were estimated from seedlings without apical buds on 3 days. At least 6 different leaves from different seedlings were used for each treatment and data are means ± SE of three replicates. Bars with different letters indicate significant difference at *P* < 0.05 (Leas significant difference test).

**Additional file 4 Figure S4.** Images of chlorophyll fluorescence in leaves of alfalfa seedlings with apical buds grown in 1.5 mM Ca(NO_3_)_2_ medium (pH 4.5) containing 0 μM AlCl_3_ (pH 4.5), 100 μM AlCl_3_ (pH 4.5 + Al), 100 μM AlCl_3_ and 50 μM ZnCl_2_ (pH 4.5 + Al + Zn), 100 μM AlCl_3_ and 6 mg L^− 1^ IAA (foliar spray) (pH 4.5 + Al + IAA) or 100 μM AlCl_3_ and 50 μM ZnCl_2_ and 6 mgL^− 1^ IAA (foliar spray) (pH 4.5 + Al + Zn + IAA) on 3 days. At least 6 different leaves from different seedlings were used for each treatment with the similar results.

**Additional file 5 Figure S5.** Effects of Zn and IAA on minimum saturating irradiance (I_*k*_) in PSI (a) and PSII (b) of seedlings with apical buds under Al stress. At least 6 different leaves from different seedlings were used for each treatment and data are means ± SE of three replicates. Values followed by different letters are significantly different at *p* ≤ 0.05 (Leas significant difference test).

**Additional file 6 Table S1.** Genes with differential expression in alfalfa leaves between normal seedlings and apical buds removed seedlings.

**Additional file 7 Table S2.** Differential expression gens involved in energy production and conversion are identified with a FDR < 0.05 and log2|FC| > =1 from leaves of normal seedlings and buds removed seedlings.

## Data Availability

The datasets supporting the conclusions of this research and materials used in this research are available by contacting with the corresponding author (anyuan@sjtu.edu.cn). The transcriptome data are shown in additional files Table S1 and Table S2.

## Abbreviations

P700/P700^+^: PSI reaction center chlorophyll dimer/oxidized form of primary electron donor of PSI
PSI and PSII: Photosystem I and II, respectively
Y(I) and Y(II): The quantum yield of PSI and PSII, respectively
Y(ND) and Y(NA): The quantum yield of non-photochemical energy dissipation in PSI owing to a shortage of electron donors[Y(ND)] or electron acceptors [Y(NA)]
Y(NPQ): The quantum yield of regulated energy dissipation in PSII
Y(NO): The quantum yield of non-regulated energy dissipation in PSII
ETRI and ETRII: Photosynthetic electron flow through PSI and PSII, respectively
*pmf*: Proton motive force
ΔpH: Trans-thylakoid pH difference or proton gradient
Pn: Net photosynthetic rate
g_H_^+^: Proton efflux rate
I_*k*_: Minimum saturating irradiance
O_2_^−^: Superoxide anion
NDH: NADH dehydrogenase-like (complex)
ALDH: Aldehyde dehydrogenase
ADH: Alcohol dehydrogenase
PEP: Phosphoenolpyruvate
BBE: Berberine bridge enzyme
