# Supplementary figures and images for: Interaction of zinc and IAA alleviate aluminum-induced damage on photosystems via promoting proton motive force and reducing proton gradient in alfalfa
Source: BMC Plant Biol. 2020 Sep 18;20:433. doi: 10.1186/s12870-020-02643-6 (PMC7501636; doi:10.1186/s12870-020-02643-6)

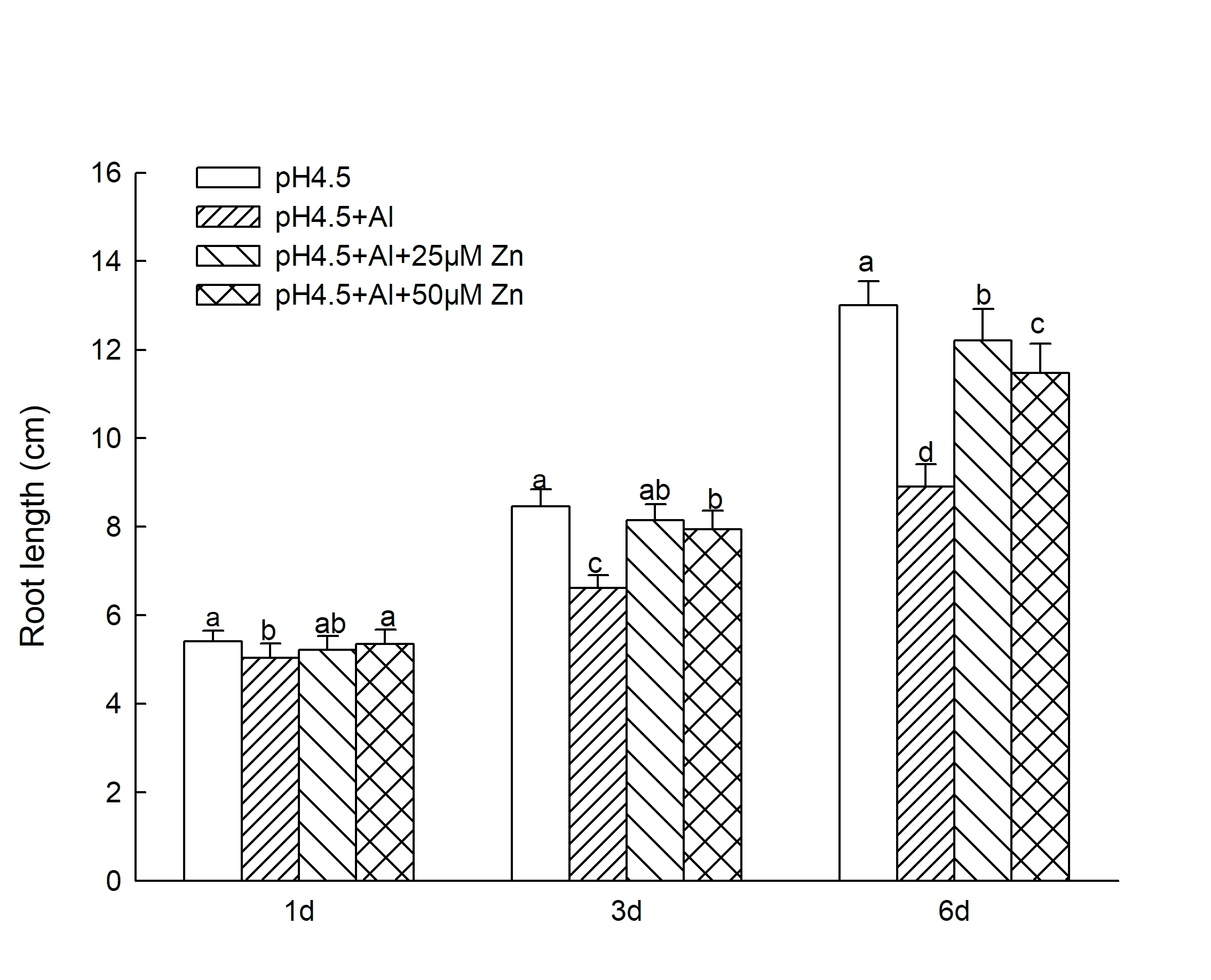

Supplement: Supplementary file 1 — Additional file 1 Figure S1. Root length of alfalfa seedlings grown in 1.5 mM Ca(NO3)2 medium (pH 4.5) containing 0 μM AlCl3 (pH 4.5), 100 μM AlCl3 (pH 4.5 + Al), 100 μM AlCl3 and 25 μM ZnCl2 (pH 4.5 + Al + 25 μM Zn), or 100 μM AlCl3 and 50 μM ZnCl2 (pH 4.5 + Al + 50 μM Zn) on days 1, 3 and 6. Data are means ± SE of three replicates from three independent experiments. Bars with different letters in the same day indicate significant difference at P < 0.05 (Leas significant difference test). [file 12870_2020_2643_MOESM1_ESM.tif]

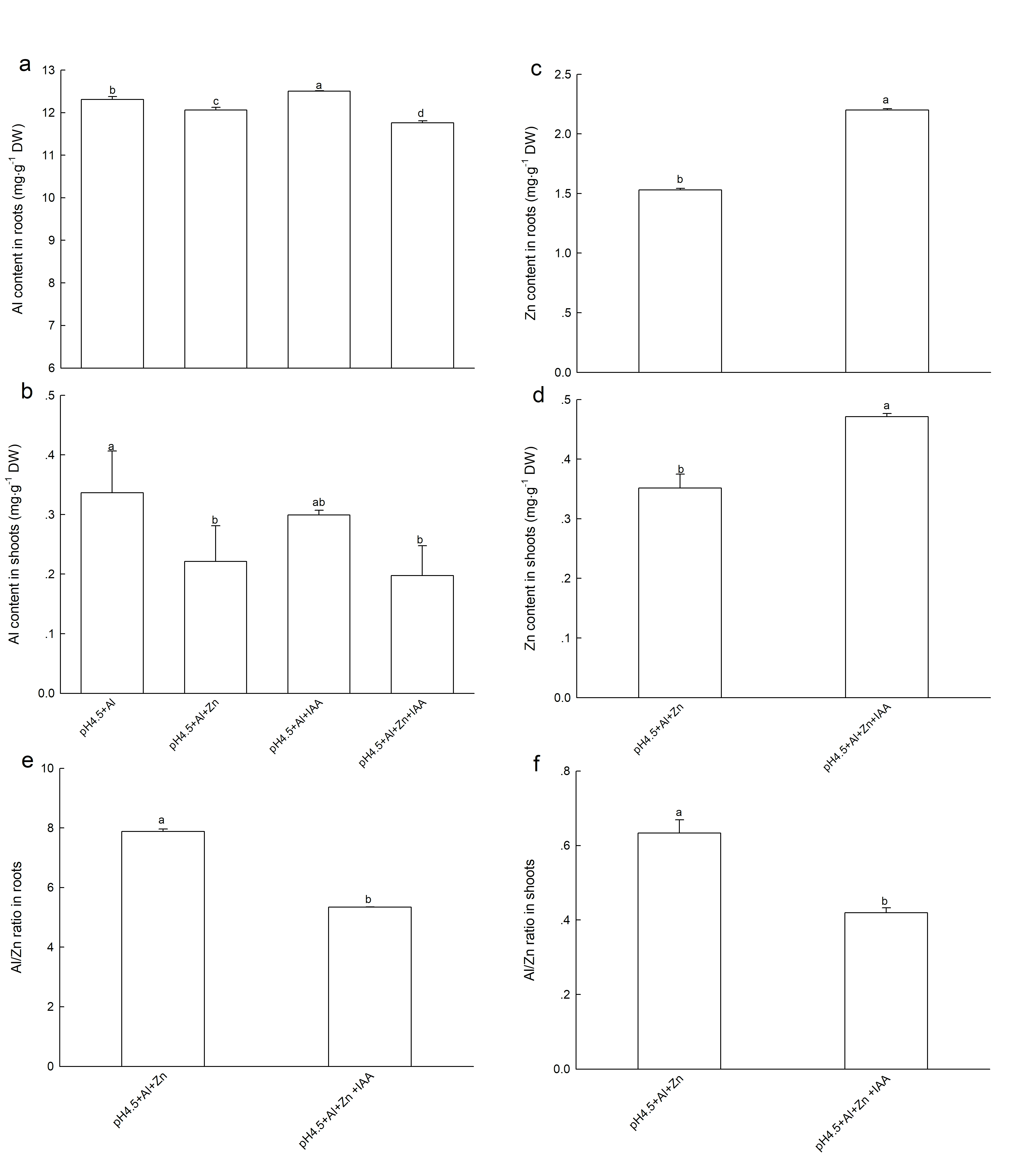

Supplement: Supplementary file 2 — Additional file 2 Figure S2. Al contents in roots (a) and shoots (b), Zn contents in roots (c) and shoots (d) and Al/Zn ratio in roots (e) and shoots (f) of alfalfa seedlings with apical buds grown in 1.5 mM Ca(NO3)2 medium (pH 4.5) containing 0 μM AlCl3 (pH 4.5), 100 μM AlCl3 (pH 4.5 + Al), 100 μM AlCl3 and 50 μM ZnCl2 (pH 4.5 + Al + Zn), 100 μM AlCl3 and 6 mg L− 1 IAA (foliar spray) (pH 4.5 + Al + IAA) or 100 μM AlCl3 and 50 μM ZnCl2 and 6 mg L− 1 IAA (foliar spray) (pH 4.5 + Al + Zn + IAA) on 3 days. Data are means ± SE of three replicates from three independent experiments. Bars with different letters indicate significant difference at P < 0.05 (Leas significant difference test). [file 12870_2020_2643_MOESM2_ESM.tif]

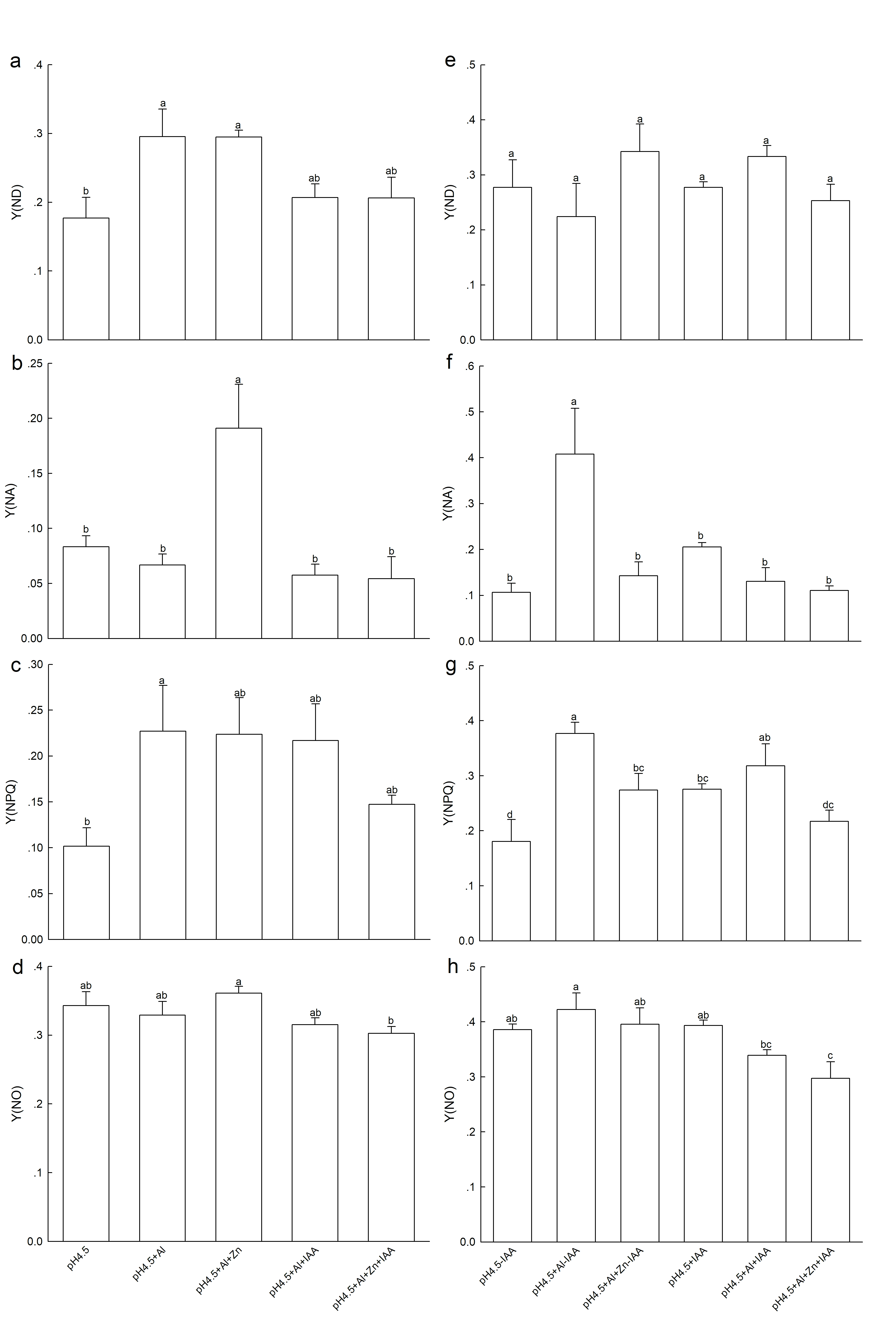

Supplement: Supplementary file 3 — Additional file 3 Figure S3. Light intensity dependence of photosynthetic quantum yields of Y(ND) and Y(NA) in PSI and Y(NPQ) and Y(NO) in PSII in leaves of alfalfa seedlings with or without apical buds. Five treatments in the seedlings with apical buds are as Fig.3, and seedlings without apical buds are grown in 1.5 mM Ca(NO3)2 medium (pH 4.5) and treated with or without spraying IAA (pH 4.5-IAA, pH 4.5 + IAA), 100 μM AlCl3 with or without spraying IAA (pH 4.5 + Al-IAA, pH 4.5 + Al + IAA) and 100 μM AlCl3 and 50 μM ZnCl2 with or without spraying IAA (pH 4.5 + Al + Zn-IAA, pH 4.5 + Al + Zn + IAA). The (a) Y(ND), (b) Y(NA), (c) Y(NPQ) and (d) Y(NO) were estimated from seedlings with apical buds, and (e) Y(ND), (f) Y(NA), (g) Y(NPQ) and (h) Y(NO) were estimated from seedlings without apical buds on 3 days. At least 6 different leaves from different seedlings were used for each treatment and data are means ± SE of three replicates. Bars with different letters indicate significant difference at P < 0.05 (Leas significant difference test). [file 12870_2020_2643_MOESM3_ESM.tif]

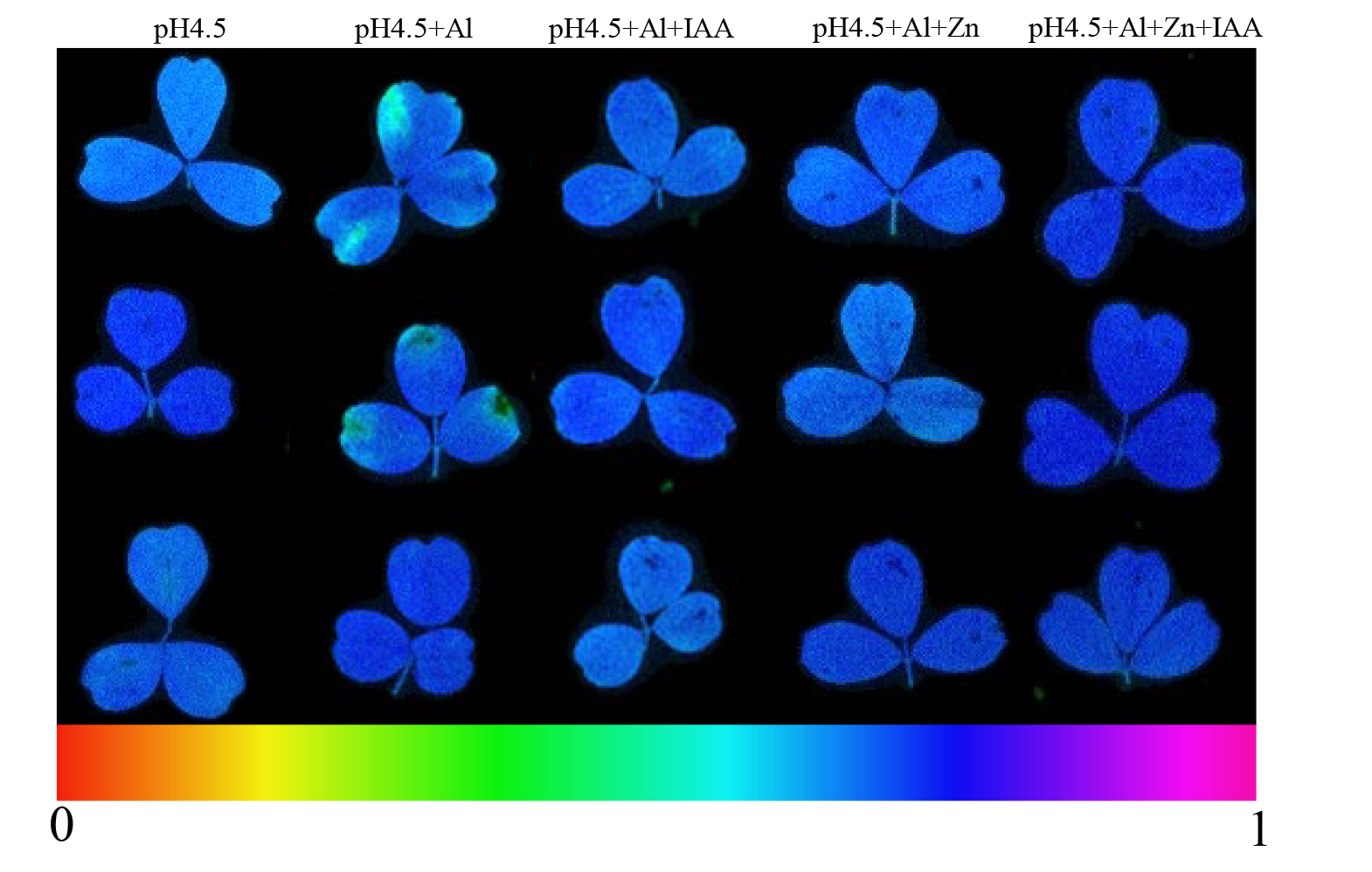

Supplement: Supplementary file 4 — Additional file 4 Figure S4. Images of chlorophyll fluorescence in leaves of alfalfa seedlings with apical buds grown in 1.5 mM Ca(NO3)2 medium (pH 4.5) containing 0 μM AlCl3 (pH 4.5), 100 μM AlCl3 (pH 4.5 + Al), 100 μM AlCl3 and 50 μM ZnCl2 (pH 4.5 + Al + Zn), 100 μM AlCl3 and 6 mg L− 1 IAA (foliar spray) (pH 4.5 + Al + IAA) or 100 μM AlCl3 and 50 μM ZnCl2 and 6 mgL− 1 IAA (foliar spray) (pH 4.5 + Al + Zn + IAA) on 3 days. At least 6 different leaves from different seedlings were used for each treatment with the similar results. [file 12870_2020_2643_MOESM4_ESM.tif]

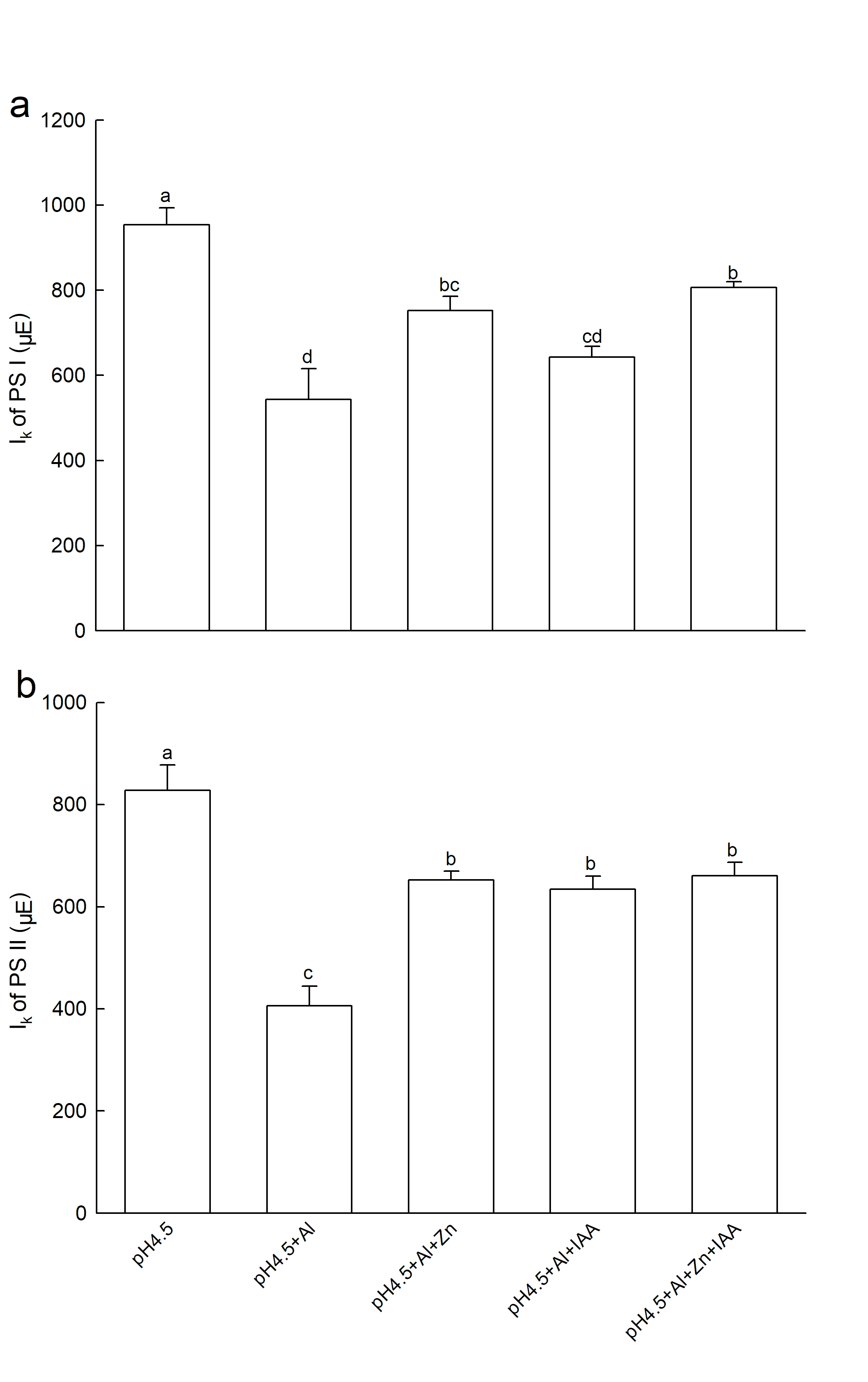

Supplement: Supplementary file 5 — Additional file 5 Figure S5. Effects of Zn and IAA on minimum saturating irradiance (Ik) in PSI (a) and PSII (b) of seedlings with apical buds under Al stress. At least 6 different leaves from different seedlings were used for each treatment and data are means ± SE of three replicates. Values followed by different letters are significantly different at p ≤ 0.05 (Leas significant difference test). [file 12870_2020_2643_MOESM5_ESM.tif]
